# Immunomodulation resulting of helminth infection could be an opportunity for immunization against tuberculosis and mucosal pathogens

**DOI:** 10.3389/fimmu.2023.1091352

**Published:** 2023-03-20

**Authors:** Kai Ling Chin, Luis Fonte, Boon Huat Lim, Maria E. Sarmiento, Armando Acosta

**Affiliations:** ^1^ Department of Biomedical Sciences, Faculty of Medicine and Health Sciences, Universiti Malaysia Sabah, Kota Kinabalu, Sabah, Malaysia; ^2^ Borneo Medical and Health Research Centre, Faculty of Medicine and Health Sciences, Universiti Malaysia Sabah, Kota Kinabalu, Sabah, Malaysia; ^3^ Department of Parasitology, Institute of Tropical Medicine “Pedro Kourí”, Havana, Cuba; ^4^ School of Health Sciences, Universiti Sains Malaysia, Kubang Kerian, Kelantan, Malaysia

**Keywords:** helminths, tuberculosis, host immune response, diagnosis, treatment, vaccine

## Introduction

Approximately, 10 million new tuberculosis (TB) cases were reported in 2020, of which 12% were among children (1). It is estimated that one-quarter of the world population (around 2 billion) is latently infected by *Mycobacterium tuberculosis* (Mtb) and 5-10% of these individuals will develop active TB (ATB) (1). Infections caused by soil-transmitted helminths (STH) and schistosomes affect about 1.5 billion and 250 million people, respectively, worldwide (2). Children and pregnant women are the population groups with the highest risk of infection by those parasites (3).

TB and helminth infections are poverty-related diseases that are epidemiologically overlapped, particularly in low- and middle-income countries. In high endemic areas of helminth and TB infections, most individuals are chronically infected by one or both types of pathogens early in life. Although socioeconomic and cultural factors could contribute to this spatial overlap (4), for many authors, these are not enough (5). Today, from a more holistic perspective that takes into account the biological components of the matter too, there is evidence that helminth infections can influence the course of other infectious diseases, such as TB, malaria, and human immunodeficiency virus (HIV) infection (6–8).

Helminths are as varied as they are ubiquitous. However, in spite of their diversity, the host protective responses against helminths, which are multicellular and large organisms, primarily include immunomodulatory and wound repair mechanisms, which reduce the tissue damage that these parasites may cause as they move through body organs (6, 7). The modulation by helminths of host immune responses has relevant clinical and epidemiological consequences, i.e., increased susceptibility to some infections, inadequate responses to vaccines, and decreased frequency and intensity of allergic, autoimmune, and inflammatory diseases, among others (9).

As part of their manipulation of the host immunity, helminths induce strong helper type 2 (Th2) responses and trigger complex immune-regulatory circuits (6, 7, 9). Protection against Mtb, as occurs with other intracellular microorganisms, requires a clearly defined Th1 responses that could be down regulated by helminths if co-infection takes place (8). Cadmus et al., in a very interesting paper published recently, revised the implications of this co-infection for tuberculosis diagnosis and vaccination in Africa, where helminths-Mtb overlap is greater (10). Here, after a brief incursion in the main immunological aspects of that co-infection, we analyze some challenges and opportunities derived of it.

## Host immune response in helminth infection

Helminths are transmitted to humans *via* ingestion of eggs or larvae, penetration of the skin by larvae, or larvae entering through insect bites on the skin (11). Helminths are macropathogens that are too large to be ingested by phagocytic cells. Helminths produce excretory/secretory antigens (Ags) (ES) which modulate the innate immune cells. Dendritic cells (DCs) recognize pathogen‐associated molecular patterns (PAMPs) on ES through various pattern recognition receptors (PRRs) including Toll-like receptors (TLRs) (e.g., TLR2 and TLR4), C-type lectin receptors (CLRs), and Nucleotide-binding and oligomerization domain-like receptors (NLRs) [e.g., mannose receptor (MR) and DC-SIGN] and serve as a mediator to adaptive immunity by presenting the Ags to T cells (12). Protection against helminth infection is predominantly mediated by Th2 cells, characterized by the production of cytokines such as IL-4, IL-5, IL-10, and IL-13; antibody class-switching to produce IgE; and activation of macrophages, eosinophils, basophils, and mast cells (12, 13). These innate cells eliminate helminths by expression of high-affinity Fc receptors in eosinophils, neutrophils, and macrophages for antibody-dependent cellular cytotoxicity and IgE-mediated degranulation of mast cells (12). Also, tissue damage by helminths triggers the production of IL-25, IL-33, and thymic stromal lymphopoietin (TSLP) (14). These cytokines are also important regulators of Th2 immune response and activate group 2 innate lymphoid cells (ILC2) which produce IL-5, IL-9, and IL-13 (14). The Th2 cytokines produced by the innate cells, particularly IL-4 and IL-13, trigger macrophages differentiation to alternatively activated macrophages (AAMs), a phenotype causing down-regulation of IFN-γ–mediated processes and inhibition of proliferation of Th1 and Th17 cells (15).

During the early phase of infection, Th1, Th2, and Th17 immune responses and IgE production reduce the infectivity of the parasite (13). The parasite survival process is the result of adaptation between host and parasite, predominantly through expansion of Th2 and contraction of Th1 subpopulations during the latency stage (11, 13). During the chronic longstanding infection, a change from inflammatory Th2 phenotype to modified Th2 phenotype is characterized by increasing secretion of IL-10 and TGF-β by regulatory T cells (Tregs) and switching of inflammatory IgE to non-inflammatory IgG4 by regulatory B cells (Bregs) (16). A study showed that infection with multiple species of worms promoted accumulation of IL-10 and TGF-β and caused down modulation of Th1 and Th2 response, resulting in immune hypo-responsiveness (17). This could be one of the reasons of reduced allergy-related diseases in helminth endemic populations.

## Host immune response to tuberculosis

TB is an airborne disease spread by aerosolized particles containing Mtb. Innate defense begins at lining of the respiratory mucosa where an invasion barrier is represented by the airway epithelial cells (AECs). The receptors on AECs recognize the PAMPs on Mtb and controls the secretion of airway surface liquid (ASL) containing antibacterial agents, and presenting the antigen to mucosal‐associated invariant T cells (MAITs) to produce IFN‐γ, TNF‐α, and granzyme for Mtb clearance (18). When Mtb successfully reach the alveoli, the bacilli are ingested by alveolar macrophages and other innate immune cells including DCs and neutrophils (19). These cells recognize Mtb through various PPRs including TLRs (e.g., TLR2, TLR4, TLR8, and TLR9) (20), NLRs (e.g., NOD1, NOD2, NLRP3, and NLRC4) (19), and CLRs (e.g., MR, DC-SIGN, dectin-1, dectin-2, and Mincle) (21) and effect a variety of innate immune defense functions such as phagocytosis, autophagy, apoptosis, and inflammasome activation (19). Nevertheless, the bacilli had various immune escape mechanisms by interfering the innate immune system which enable them to survive and multiply intracellularly, including inhibition of maturation and acidification of the phagolysosome, inhibition of oxidative stress, inhibition of autophagy and apoptosis, altering recognition by structural mutations, suppression of cellular immune responses and induction of tolerance, among others (19).

The onset of adaptive immune response begins when Mtb-infected DCs migrate into the lymph nodes and prime CD4 T-cells. During the pre-immune specific phase, before the availability of activated specific cellular effectors, bacilli multiply in the lungs (22). Mtb is an intracellular pathogen that elicits protective Th1 immune responses (IFN-γ and TNF-α) against Mtb by activating the antimycobacterial mechanisms in macrophages (23). The balance between Th1 and Th17 (IL-17 and IL-22) is important to control bacterial growth and limit immunopathology because the later have been implicated in TB pathology including neutrophilic inflammation and tissue damage (22). Following the establishment of both innate and adaptive immunity, Mtb is contained in granulomas, cellular aggregates constituted by macrophages, multinucleated giant cells, epithelioid and foamy cells, granulocytes, and lymphocytes (23), in a non-replicating phase (multiplication of Mtb is halted by the immune system to prevent further progression) which is observed in 90% of Mtb infected individuals, also known as latent Mtb infection (LTBI), which is an asymptomatic and non-infectious state (24). In some LTBI people (5-10%), Mtb overcome the immune system control and multiply, macrophages in the granuloma die, and the caseous necrotic center of the granuloma liquefies and cavitates, leading to the release of Mtb into the airways, resulting in progression from LTBI to TB disease and transmission (25). Also, an immune balance between Tregs and Th17 is important to control Mtb growth without causing tissue damage and to encapsulate granuloma to limit Mtb spread. Imbalance of Tregs/Th17, i.e., favoring Tregs will cause Mtb dissemination, while favoring Th17 will cause inflammation and neutrophils recruitment, resulting in growth of the granuloma and development of TB (26).

## Immune response to helminths and *Mycobacterium tuberculosis* co-infection

In acute helminthic infections there may be activation of Th1 responses that would stimulate type 1 alveolar macrophages (involved in the early control of mycobacterial infection). Consequently, primary helminth infection could contribute to early control of mycobacterial infection (27). In correspondence with this, different studies carried out in animals co-infected show an accelerated increase in the number of alveolar type 1 macrophages, which occurs transiently, only during the acute stage of the helminthic infection (27). *In vivo* studies in mice showed that acute helminth infection increased CD4 T cells count and Th1 cytokine levels (IFN-γ) which may contribute to the augmentation of the activation and recruitment of neutrophils and alveolar macrophages for early control of mycobacterial infection (27). *In vitro* models also showed that helminth infections directly reduced/control Mtb growth in monocytes and macrophages before development of cell-mediated immunity (28).

A study by O’shea et al. showed that there is a negative association between helminth infection and LTBI-positivity, and blood from helminth-infected individuals had increase of eosinophils and greater ability to control Mtb growth, suggesting the possibility of a reduction of LTBI prevalence among individuals with helminth-induced eosinophilia (29). However, in a later stage, eosinophilia has been associated with the progression to ATB (30).

In Mtb infected patients, the chronic infection with *Strongyloides stercoralis* had significantly skewed the Th1 and Th17 immune responses during TB infection to a predominantly Th2 response with increase of Tregs. In these co-infected patients, the production of Th1 (INF-γ, TNF-α, and IL-12) and Th17 (IL-17A and/or IL-17F) cytokines were decreased, while Th2 (IL-4 and IL-5) and Tregs (TGF-β) cytokines were increased (31). The Th2, Tregs, and immunomodulatory cytokines (mainly IL-10, and TGF-β) produced during helminth infection may act as inhibitors of Th1 responses (reduced IFN-γ and TNF-α) required for Mtb infection control and increased the risk of reactivation in LTBI (10).

The helminth-Mtb co-infection compromised the immune response of monocytes, i.e., reduced frequencies of monocytes expressing CD80/CD86 (molecules that interact with CD28 and CTLA-4 for T-cell activation), elevated M2 polarization (anti-inflammatory monocytes/macrophages), reduced monocyte cytokines IL-1β, TNF-α, IL-6, and IL-12 production, increased IL-10 production, and diminished T-cell activation (32). In addition, IL-10 impaired macrophage activities by delaying phagosome maturation and inhibiting the expression of IFN-γ-induced antimycobacterial effector molecules (33). Interestingly, following anti-helminthic treatment, the altered monocyte functions are restored (32) and Th1 and Th17 cytokines increased, and Th2 and Tregs cytokines decreased (31).

In helminth-infected TB patients, TLR2 and TLR9 in PBMCs and the pro-inflammatory cytokine (IL-1β, IL-6, IFN-γ, IL-12, and TNF-α) responses to TLR2 and TLR9 ligands were significantly reduced (34). The responsiveness to these ligands were restored upon anti-helminthic therapy (34). Also, the downregulation of CLRs by IL-4 and IL-13 in helminth-infected patients, reduced amounts of pro-inflammatory cytokines like IL-6, IL-1, or G-CSF, impaired phagocytosis, and Th1/Th17 differentiation (33).

In a mice model, it was observed that the helminth-induced Th2 environment and increased IL-4, caused accumulation of AAMs. Attenuation of the nitric oxide production by these cells compromised the ability to kill Mtb, resulting in intracellular survival and multiplication of the bacilli (35). Also, helminth co-infection caused accumulation of high arginase-1 expressing macrophages in the lung forming type 2 granulomas and resulting in lung fibrosis, mucous plug formation, and exacerbated inflammation that damage the lung (36).

The helminth-Mtb co-infected patients also present more advanced TB. In individuals co-infected with *S. stercoralis* and pulmonary TB there is an increased risk of bilateral lung lesions and cavitation, and higher plasma levels of matrix metalloproteinases (MMPs) (37). Another study on *S. stercoralis* and tuberculous lymphadenitis (extrapulmonary TB) co-infection showed elevated levels of MMPs and tissue inhibitor of metalloproteinases (TIMPs) (38). The increased secretion of MMPs increased inflammatory responses in the host because it may cause extracellular matrix degradation (i.e., basement membrane disintegration, proteolytic cleavage of tissue matrix, and collagen breakdown), contributing to the dissemination of TB infection, and recruitment of leucocytes which leads to necrosis and cavitation (38).

At least, three additional studies evidence that the modulation of Th2 and Tregs immune responses by helminths could downregulate the Th1 and Th17 immune responses against Mtb infection and lead to the progression of LTBI to ATB and more serious forms of ATB associated to therapeutic failure: (i) endogenous reactivation of LTBI has been associated with increased production of IL-10 and TGF-β by circulating monocytes and possibly Tregs (39) and (ii) in patients with LTBI, co-infection with helminths (filariae and hookworms) can diminish Mtb-specific Th1 (IL-12 and IFN-γ) and Th17 (IL-23 and IL-17) responses and may predispose toward the development of ATB (34, 40, 41). Together with those immunological evidences that reinforce the opinion that helminth co-infection reduces the host resistance to Mtb infection, it is necessary to mention two epidemiological researches with divergent results: a study in Ethiopia found a greater risk of ATB in intestinal helminth co-infected individuals (42), whereas an investigation in India on individuals infected with intestinal and filarial helminths did not encounter significant progression from LTBI to ATB (43). Analyzing the divergence from a more holistic perspective, Aranzamendi et al. advanced the opinion that it may be the consequence of the interaction of factors such as species of helminth involved, parasite load, and infection chronicity (44).

## Impact of helminth infections on TB diagnosis, treatment, and vaccination

In TB endemic countries, sputum smear microscopy is primarily used for diagnosis of PTB. It is suggested that helminth infection alters the clinical presentation in TB patients, but the findings are inconclusive. Kumar et al. (2020) showed that *S. stercoralis-*infected TB patients had greater bacterial burden and lungs cavitation (37), but Mhimbira et al. (2017) showed lower rate of sputum smear positivity and less lungs cavitation only in *Schistosoma mansoni*-infected TB patients, not in other helminth infections (45). Therefore, a new area worth to be explored is to determine the helminth species-specific impact on immune response and its impact on diagnosis and clinical presentation.

Currently, there are only two methods in use for LTBI screening, i.e., tuberculin skin test (TST) which measure delayed-type hypersensitivity (DTH), T-cell responses to purified protein derivative (PPD), and interferon-gamma (IFN-γ) release assays (IGRAs), which measure the T-cell release of IFN-γ following stimulation with Mtb antigens (ESAT-6 and CFP-10), both tests are consequences of immune responses Th1 mediated. Helminth infections which induce Th2 responses (anti-Th1) and a hypo-responsive state indirectly reduce the DTH responses (46) and altered the cytokine production resulting in indeterminate IGRA results (47). A study by Toulza et al. (2015) showed that helminth-infected patients had significant reduction of CD4^+^IFN-γ^+^ T-cells after stimulation with PPD and ESAT-6/CFP10, and the effects were reversed after anti-helminthic treatment (48). Another study showed that high IgE production induced by helminths had significantly reduced the TST positivity in children but the effect is weakened with increasing age (49). Considering the negative influence of the Th2 response associated to helminth infections with an inhibitory effect on Th1 responses, and the reports of Th2 cytokine responses, such as IL-10, to Mtb antigens in helminth infected TB patients (50), the supplementation of the current IGRA tests with the determination of Th2 cytokines, such as IL-10 could be explored to increase the performance of these tests in helminth endemic populations.

In addition to a negative impact on anti-Mtb immunity, intestinal helminth infection has shown to have a negative influence on clinical response to anti-TB therapy. As an efficient clinical response to Mtb treatment is dependent upon an effective Th1 response, the more delayed clinical evolution in Mtb-helminth co-infected patients in association with increased IL-10 levels may indicate that helminth infection in TB patients may tilt their cytokine profile towards a Th2 response (50).

In a prospective hospital-based study, it was observed that besides older age (>40 years old) and diabetes mellitus, hookworm infection could cause high therapeutic failure in PTB (51). Another research showed that treatment with anti-parasites drugs, such as albendazole, could reduce the eosinophils counts and IL-10 level (52). This is indicative that timely diagnosis and treatment of intestinal helminth infection may be important for a successful response to anti-TB treatment. Some classical anti-helminthic drugs, such as avermectins, mefloquine, niclosamide, nitazoxanide, nitroimidazoles, pyronaridine, and auranofin, have anti-TB activity too (53). In future, repurposing these anti-helminthic drugs could be exploited for complementary anti-TB treatment.

Bacille Calmette-Guérin (BCG), a strong Th1 inducer, is a vaccine for protection against TB, recommended for neonates and infants from countries with high endemicity of TB (54). A study by Elias et al. (2008) showed that chronic helminths infected populations had poor immune response to BCG vaccination due to impaired Th1 responses (reduced IFN-γ and IL-12) and enhanced Tregs responses (increased TGF-β) (55). These responses were reversed by anti-helminthic treatment (55) and monocytes were able to respond optimally to TB antigenic stimulation (32), suggesting that deworming may be necessary in helminth endemic countries to restore immune responsiveness and improve the efficacy of BCG.

Secretory IgA (sIgA) serves as first-line defense in protecting intestinal, respiratory, and urogenital mucosal epithelia and maintenance of mucosal homeostasis. It is known that TGF-β production/presence is associated to a switch for IgA production (56). Therefore, the cytokine environment characteristic of helminth infection (high TGF-β) could be conductive to produce sIgA mucosal responses, which could be considered for the prioritization of development of mucosal vaccines for TB and other microorganisms in populations with high helminth endemicity, to complement the “classical” Th1 inducing vaccines in use and evaluation for different diseases. This kind of vaccines could be a valuable tool in the prevention of primary infection, avoiding the microorganism entrance, the subsequent development of acute and chronic infections, and transmission (57, 58). The use of such vaccines in children, during the first year of life could be of paramount importance to prevent primary infection with multiple infectious agents. In this regard, it has been reported that the presence of helminth infection during pregnancy could bias the immune response of the child toward an IgA response to oral vaccines during the first year of life, compared to children born from helminth uninfected mothers. It has been hypothesized that the placental transfer of TGF-β and IL-10 during pregnancy, and through breast milk during lactation “educate” the immune response of the child promoting mucosal IgA responses (59). At the same time, several helminth infections are associated with eosinophils presence and activation, which correlated with the evidences of the role of eosinophils in the production and maintenance of IgA plasma cells (60).

Considering the route of entry of Mtb *via* inhalation, mucosal vaccines could represent a promissory alternative for TB vaccination (61, 62). Multiple experimental results, including non-human primates (NHP), demonstrated the protective effect of mucosal TB vaccine candidates using intranasal and aerosol routes of administration, different technological vaccine platforms, and different schemes of immunization such as prime or as BCG booster (62–68). In line with it, some mucosal prophylactic vaccines for TB have been tested in human clinical trials (61). Experimental studies using IgA antibody formulations provide additional support of the potential of mucosal immunization for TB protection, in this regard, it has been reported that the mucosal administration of secretory IgA monoclonal antibodies directed to Mtb HspX surface antigen and human secretory IgA purified from colostrum before Mtb challenge in mice induced a significant decrease of bacterial burden and histopathological lesions in lungs compared to untreated controls (69, 70).

Besides TB and other infectious diseases gaining access by the respiratory, gastrointestinal, genitourinary and other mucosal sites, the development of mucosal vaccines against helminths is another possibility in helminths endemic areas, in this regard, the protective role of SIgA against helminth infections after mucosal vaccination in different animal models have been reported (60). The impact of SIgA in protection was associated to the presence of lower parasitic loads, fecal and female eggs count, and worm length, which is consistent with *in vitro* reported activities of SIgA from immunized animals, such as antibody-dependent cellular cytotoxicity (ADCC) of eosinophils, neutralization of helminth excretory/secretory products and the attachment to different helminth stages (60).

## Conclusion

In high endemic areas of helminth and TB infections, most individuals are chronically infected by one or both types of pathogens early in life. In chronic helminth infection, the modulation of Th2 and Tregs immune responses by those parasites could downregulate the Th1 and Th17 immune responses against Mtb infection and leads to the progression of LTBI to ATB and more serious forms of active TB associated to therapeutic failure. The helminth immune modulation could also cause indeterminate results in LTBI diagnostic tests and poor immunogenicity of BCG vaccination. From clinical and epidemiological perspectives, it is necessary to consider that the immunomodulation in co-infected patients could be reversed through anti-helminthic treatments, suggesting with an additional argument that deworming programs are necessary in the countries with high burden of helminth and TB infections. Looking ahead, the immune environment of high TGF-β level associated to helminth infections suggests the promissory use of TB mucosal immunization as an alternative, or complement, to “classical” Th1 inducing vaccines in co-infection settings.

## Author contributions

All authors listed have made a substantial, direct, and intellectual contribution to the work, and approved it for publication.
